# Development of Yellow Rust-Resistant and High-Yielding Bread Wheat (*Triticum aestivum* L.) Lines Using Marker-Assisted Backcrossing Strategies

**DOI:** 10.3390/ijms26157603

**Published:** 2025-08-06

**Authors:** Bekhruz O. Ochilov, Khurshid S. Turakulov, Sodir K. Meliev, Fazliddin A. Melikuziev, Ilkham S. Aytenov, Sojida M. Murodova, Gavkhar O. Khalillaeva, Bakhodir Kh. Chinikulov, Laylo A. Azimova, Alisher M. Urinov, Ozod S. Turaev, Fakhriddin N. Kushanov, Ilkhom B. Salakhutdinov, Jinbiao Ma, Muhammad Awais, Tohir A. Bozorov

**Affiliations:** 1 Institute of Genetics and Plant Experimental Biology, Academy of Sciences of the Republic of Uzbekistan, Tashkent 111208, Uzbekistan; meliev.sodir@mail.ru (S.K.M.); fmelikuziev23@gmail.com (F.A.M.); ilhamaytenov@gmail.com (I.S.A.); murodovasojida1433@gmail.com (S.M.M.); xalillaevagavhar@gmail.com (G.O.K.); genetik8181@mail.ru (B.K.C.); laylobio@gmail.com (L.A.A.); ozodturaev@gmail.com (O.S.T.); fakhriddinkushanov@gmail.com (F.N.K.); tohirbozorov@yahoo.com (T.A.B.); 2 Department of Medicine, Alfraganus University, Tashkent 100190, Uzbekistan; a.orinov@afu.uz; 3 Research Institute of Plant Genetic Resources, National Center for Knowledge and Innovation in Agriculture, Tashkent 100180, Uzbekistan; 4 Department of Genetics, National University of Uzbekistan, Tashkent 100174, Uzbekistan; 5 Center of Genomics and Bioinformatics, Academy of Sciences of the Republic of Uzbekistan, Tashkent 111215, Uzbekistan; ilkhom.salakhutdinov@genomics.uz; 6 State Key Laboratory of Desert and Oasis Ecology, Key Laboratory of Ecological Safety and Sustainable Development in Arid Lands, Xinjiang Institute of Ecology and Geography, Chinese Academy of Sciences, Urumqi 830011, China; majinbiao@ms.xjb.ac.cn (J.M.); awaismuhammad@nwafu.edu.cn (M.A.)

**Keywords:** wheat, *Triticum*, marker-assisted backcross breeding, advanced backcross breeding lines (ABLs), disease resistance, *Puccinia striiformis* f. sp. *tritici* (*P. striiformis*), SSR markers, yellow rust, *Yr10*, *Yr15*

## Abstract

The fungal pathogen *Puccinia striiformis* f. sp. *tritici*, which causes yellow rust disease, poses a significant economic threat to wheat production not only in Uzbekistan but also globally, leading to substantial reductions in grain yield. This study aimed to develop yellow rust-resistance wheat lines by introgressing *Yr10* and *Yr15* genes into high-yielding cultivar Grom using the marker-assisted backcrossing (MABC) method. Grom was crossed with donor genotypes Yr10/6*Avocet S and Yr15/6*Avocet S, resulting in the development of F_1_ generations. In the following years, the F_1_ hybrids were advanced to the BC_2_F_1_ and BC_2_F_2_ generations using the MABC approach. Foreground and background selection using microsatellite markers (Xpsp3000 and Barc008) were employed to identify homozygous Yr10- and Yr15-containing genotypes. The resulting BC_2_F_2_ lines, designated as Grom-Yr10 and Grom-Yr15, retained key agronomic traits of the recurrent parent cv. Grom, such as spike length (13.0–11.9 cm) and spike weight (3.23–2.92 g). Under artificial infection conditions, the selected lines showed complete resistance to yellow rust (infection type 0). The most promising BC_2_F_2_ plants were subsequently advanced to homozygous BC_2_F_3_ lines harboring the introgressed resistance genes through marker-assisted selection. This study demonstrates the effectiveness of integrating molecular marker-assisted selection with conventional breeding methods to enhance disease resistance while preserving high-yielding traits. The newly developed lines offer valuable material for future wheat improvement and contribute to sustainable agriculture and food security.

## 1. Introduction

Wheat (*Triticum* spp.) is one of the most widely cultivated staple crops worldwide. Among the major constraints to wheat productivity are fungal diseases, including yellow rust (also known as stripe rust), which is one of the most widespread and damaging. Yellow rust, caused by *Puccinia striiformis* f. sp. *tritici* (*P. striiformis*), is prevalent in cool and humid climates. Over the past few decades, new, aggressive, and genetically diverse *P. striiformis* populations adapted to higher temperatures have emerged, significantly weakening the resistance of many wheat cultivars [1,2,3]. Currently, approximately 88% of global wheat varieties are susceptible to *P. striiformis*, with an estimated annual yield loss of 5.47 million tons attributed to yellow rust [4,5,6]. Recent studies have shown that infection by this pathogen can result in yield reductions ranging from 20% to 40% [7,8], and in severe cases, losses may reach up to 100% in susceptible cultivars [1].

In Uzbekistan, wheat, particularly common or bread wheat (*T. aestivum* L.), plays a vital role in national food security. Consequently, ongoing research in agrotechnological, physiological, and molecular breeding efforts aim to enhance productivity and grain quality under both biotic and abiotic stress conditions [9,10,11,12,13]. Among the most effective and sustainable strategies to control yellow rust is the development and deployment of resistant cultivars, which requires a detailed understanding of the genetic and molecular basis of resistance.

Marker-assisted selection (MAS) has emerged as a powerful and reliable approach for improving disease resistance in wheat breeding. It allows for the accurate identification and introgression of target resistance genes, even at early developmental stages, and facilitates the pyramiding of multiple favorable alleles into elite cultivars via backcrossing [14,15,16]. Compared to traditional phenotypic selection, MAS enables more rapid, precise, and cost-effective breeding outcomes. Furthermore, it overcomes limitations associated with environment-dependent expression of resistance and allows for the efficient combination of multiple resistance genes within a single genotype [17,18].

The *Yr10* gene, a dominant resistance gene to *P. striiformis*, was first identified in the wheat line PI 178383 and cultivar Moro [19]. It has since been reported in several wheat-growing countries, including China [20], India [21], Pakistan [22], Iran [23], and the United States [24], and has shown effectiveness against all known *P. striiformis* races in Kazakhstan [25]. The SSR marker Xpsp3000 is tightly linked to *Yr10* and widely used in MAS pipelines. The donor line Yr10/6*Avocet S, developed by introgressing *Yr10* into the susceptible Avocet S background, is one of the well-characterized advanced backcross breeding lines (ABLs) used for both phenotyping and gene pyramiding.

Similarly, the *Yr15* gene, also dominant, was identified in the wild emmer wheat accession G-25 (*Triticum dicoccoides* Korn.) [26]. It provides broad-spectrum resistance to multiple *P. striiformis* races and has been validated in diverse environments, including Central Asia. The donor line Yr15/6*Avocet S carries *Yr15* in a similar genetic background as Yr10/6*Avocet S, making it ideal for comparative genetic studies. The *Yr15* gene is closely linked to SSR markers Barc008 and Xgwm413, which were identified by several researchers [27,28,29,30].

Therefore, the main objective of this study was to introgress the *Yr10* and *Yr15* resistance genes into the high-yielding cultivar Grom via the MABC method, and to develop ABLs carrying these genes. The findings demonstrate the successful development of yellow rust-resistant ABLs that retained the superior agronomic traits of Grom, with no adverse effect on yield performance. This work illustrates the effectiveness of integrating molecular and conventional breeding strategies to generate disease-resistant and agronomically superior wheat cultivars.

## 2. Results

### 2.1. Development of Backcross Combinations Carrying Yellow Rust Resistance Genes

The development of ABLs conferring resistance to yellow rust in the high-yielding wheat cultivar Grom was achieved through marker-assisted introgression of the *Yr10* and *Yr15* resistance genes. These genes were sourced from the isogenic lines Yr10/6*Avocet S and Yr15/6*Avocet S, respectively. The initial crosses between the hexaploid (2n = 6x = 42) wheat cultivars Grom and the respective donors resulted in F_1_ hybrid plants carrying putative resistance alleles.

To confirm the presence of the target genes, F_1_ hybrids were screened using the SSR markers Xpsp3000 and Barc008, which are tightly linked to *Yr10* and *Yr15*, respectively. All F_1_ individuals were identified as heterozygous for the respective target loci, indicating successful initial gene transfer. Subsequent backcrossing of F_1_ hybrids to the recurrent parent cv. Grom resulted in two BC_1_F_1_ populations. The progeny from these hybridizations displayed variation in seed morphology, ranging from shriveled to normally filled. The PCR screening analysis revealed that, in the *Yr10*-targeted two population, eight out of seventeen BC_1_F_1_ plants were heterozygous for *Yr10* as determined by Xpsp3000. Similarly, in the *Yr15* population, fifteen out of thirty-two BC_1_F_1_ hybrids were identified as heterozygous for the *Yr15* genes, using Barc008 marker.

Further refinement involved selecting eight and fifteen BC_1_F_1_ plants from the Yr10/6*Avocet S and Yr15/6*Avocet S lines, respectively, all of which were phenotypically similar to the recurrent parent cv. Grom. The most promising plant from each donor line was subsequently backcrossed with Grom to generate the BC_2_F_1_ populations. Within this population, 57 out of 124 BC_2_F_1_ genotypes were identified as heterozygous carriers of the *Yr10* gene, and 51 out of 110 hybrids were heterozygous for the *Yr15* gene, as determined by SSR markers. A similar marker-assisted selection procedure was applied to the BC_2_F_1_ plants, which were then self-pollinated to produce the BC_2_F_2_ generation. In the BC_2_F_2_ population, 23 plants were confirmed to be homozygous for the *Yr10* gene, and 29 plants were homozygous for the *Yr15* gene as detailed in Table 1.

**Figure 1:**
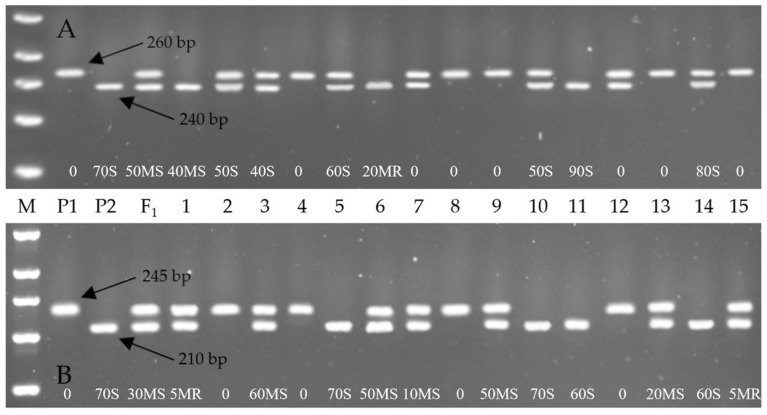
Representative agarose gel electrophoresis profiles showing foreground selection for the yellow rust resistance genes *Yr10* and *Yr15* using gene-linked SSR markers. (**A**) Amplification with Xpsp3000, a marker linked to the *Yr10* gene. (**B**) Amplification with Barc008, a marker linked to the *Yr15* gene. M: Molecular weight marker; P1: donor parent (Yr10/6*Avocet S in (**A**); Yr15/6*Avocet S in (**B**)); P2: recurrent parent (Grom); F_1_: First generation hybrid; Lanes 1–15: BC_2_F_2_ plants.

**Figure 2:**
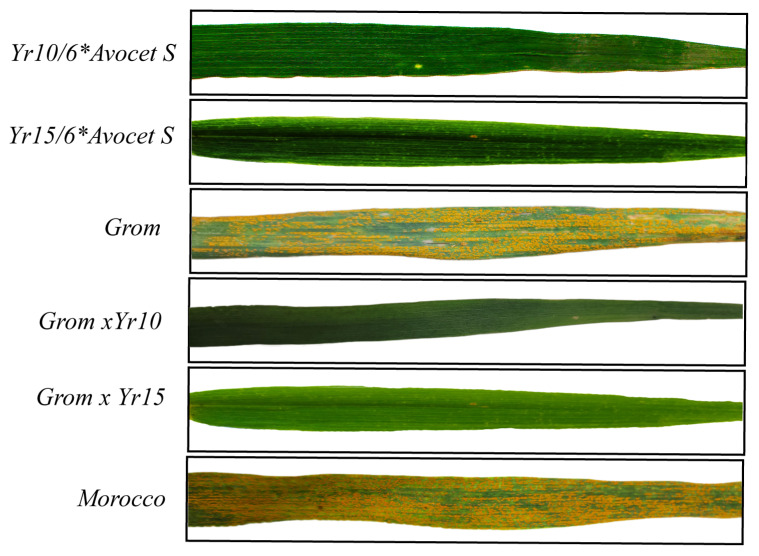
Phenotypical evaluation of yellow rust resistance in wheat leaves under artificial infection conditions. Image displays representative leaves of parental donor lines Yr10/6*Avocet S (harboring the *Yr10* gene) and Yr15/6*Avocet S (harboring the *Yr15* gene); the susceptible high-yielding recurrent parent Grom; newly developed ABLs, Grom × Yr10 and Grom × Yr15; and the susceptible check cultivar Morocco. Clear differences in disease severity are observed, with ABLs showing resistance comparable to the donor lines, while Grom and Morocco exhibit extensive stripe rust symptoms.

The culmination of this meticulous foreground and background marker-assisted selection combined with phenotypic evaluation led to the successful development of ABLs in the cv. Grom genetic background, exhibiting stable resistance to yellow rust. This achievement is visually represented in Figure 1 and Figure 2, which illustrate the MABS scheme and the gel electrophoresis image of the foreground selection, respectively. The resistant wheat genotypes were selected based on a combination of molecular marker profiles and disease resistance phenotypes. Specifically, 23 BC_2_F_2_ individual plants derived from the Grom × Yr10/6*Avocet S combination exhibited homozygosity for the Yr10-linked Xpsp3000 marker, as observed in samples of 4, 8, 9, 13 and 15 (Figure 1A). Also, 29 hybrids from the Grom × Yr15/6*Avocet S combination showed homozygosity for the Yr15-linked Barc008 marker, with representative samples being 2, 4, 8 and 12 (Figure 1B).

### 2.2. Phenotyping of ABLs for Yellow Rust Resistance

The resistance of ABLs carrying the *Yr10* and *Yr15* genes was evaluated under artificial infection conditions to confirm their phenotypic response to yellow rust. The parental lines—Grom, Yr10/6*Avocet S, and Yr15/6*Avocet S, together with the ABLs and the susceptible control cultivar Morocco—were phenotyped according to the disease rating scale developed by Peterson et al. [31], at the booting, heading, and flowering growth stages.

Upon evaluation, both Grom and Morocco exhibited a susceptible infection type (IT = “S”), whereas the donor parents Yr10/6*Avocet S and Yr15/6*Avocet S showed complete resistance (IT = “0”) (Figure 2). All 23 and 29 ABLs derived from the respective BC_2_F_2_ populations (Grom-Yr10 and Grom-Yr15) also displayed homozygous resistance phenotypes (IT = “0”) across all replicates.

These results confirm that the ABLs selected via foreground markers Xpsp3000 (linked to *Yr10*) and Barc008 (linked to *Yr15*) exhibit stable and complete resistance to yellow rust, indicating the successful introgression and expression of the resistance genes in the Grom genetic background.

### 2.3. Selection of ABLs for High-Yielding Traits

**Figure 3:**
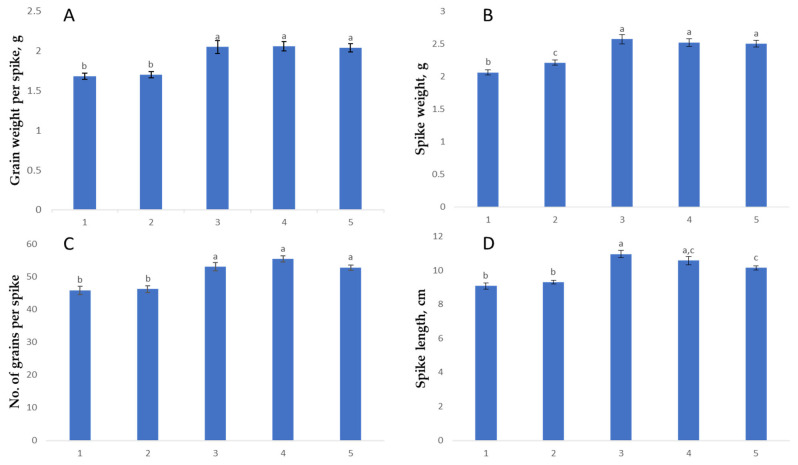
Statistical assessment of yield trait variability among samples using ANOVA and post-hoc analysis. (**A**) Grain weight per spike (g); (**B**) Spike weight (g); (**C**) Number of grains per spike; (**D**) Spike length (cm). 1: Yr10/6*Avocet S; 2: Yr15/6*Avocet S; 3: Grom; 4: Grom-Yr10; 5: Grom-Yr15. Different lowercase letters above the bars indicate significant differences between genotypes at *p* < 0.05 according to Tukey’s HSD test. Error bars represent standard errors of the means.

Following marker-assisted backcrossing, a total of 23 homozygous BC_2_F_2_ plants carrying the *Yr10* gene (from Yr10/6*Avocet S and Grom) and 29 homozygous plants carrying the *Yr15* gene (from Yr15/6*Avocet S and Grom) were identified. The selected ABLs were then evaluated for yield-related spike and grain traits, including spike length, number of spikelets per spike, number of grains per spike, and thousand kernel weight (Table 2; Figure 3).

The novelty of our research lies in the successful development of BC_2_F_2_-derived lines that not only carry the *Yr10* and *Yr15* resistance genes but also retain the desirable agronomic traits of the Grom variety. Field evaluations under natural disease pressure confirmed that the derived lines showed stable and strong resistance against stripe rust, while agronomic performance, particularly grain yield, was either comparable to or superior to the recurrent parent and commercial checks.

Spike Length: The recipient genotype Grom exhibited the highest mean spike length (10.97 ± 0.21 cm), indicating its superior growth trait compared to the donor lines. The BC_2_F_2_ line derived from Grom-Yr10 showed a mean spike length closer to Grom (10.58 ± 0.23 cm), suggesting that the introgression of Yr10/6*Avocet S may have a lesser impact on reducing spike length compared to Yr15/6*Avocet S. Both BC_2_F_2_ lines, with the maximum trait indicators, had spike lengths of 11.9 and 13.0 cm, respectively, matching Grom at 13.0 cm, whereas Yr10/6*Avocet S and Yr15/6*Avocet S reached 11.0 cm in spike length.

Spike Weight: Grom led again in spike weight (2.57 ± 0.07 g), reinforcing the recipient’s superior agronomic traits. Among the BC_2_F_2_ lines, Grom-Yr10 achieved a spike weight (2.52 ± 0.07 g) that was closer to Grom, outperforming Grom-Yr15 (2.5 ± 0.05 g), which aligns with the trend observed in spike length. The BC_2_F_2_ lines Grom-Yr10 and Grom-Yr15 had maximum spike weights of 3.23 g and 2.92 g, respectively, while Grom reached 3.18 g. The donor parents Yr10/6*Avocet S and Yr15/6*Avocet S achieved spike weights of 2.62 g and 2.89 g, respectively.

Grain Weight per Spike: The Grom sample exhibited the highest yield with an average grain weight of 2.06 ± 0.08 g per spike. The grain weight ranged from 1.66 g to 2.97 g, demonstrating the potential of this genotype. Following closely in the BC_2_F_2_ generation were Grom-Yr10 (2.05 ± 0.06 g) and Grom-Yr15 (2.04 ± 0.05 g). The donor varieties showed lower average values, with Yr10/6*Avocet S at 1.68 ± 0.04 g and Yr15/6*Avocet S at 1.70 ± 0.04 g. The average maximum grain mass per spike in the Grom variety was 2.97 g, while in the hybrid samples BC_2_F_2_ (Grom-Yr10) and BC_2_F_2_ (Grom-Yr15), it reached 2.82 g and 2.41 g, respectively. The donor varieties achieved maximum grain weights of 2.30 g (Yr10/6*Avocet S) and 2.36 g (Yr15/6*Avocet S), respectively.

Number of Spikelets per Spike: The recipient Grom demonstrated the highest mean number of spikelets per spike (20.17 ± 0.20), suggesting it has a denser spike structure. The BC_2_F_2_ lines Grom-Yr10 (19.88 ± 0.31) closely followed Grom, showing the minimal impact on the number of spikelets, whereas Grom-Yr15 (19.30 ± 0.29) showed a reduction. The donor parents Yr10/6*Avocet S and Yr15/6*Avocet S recorded a lower number of spikelets, 16.14 ± 0.25 and 16.28 ± 0.18 per spike, respectively. The BC_2_F_2_ lines Grom-Yr10 and Grom-Yr15 exhibited a maximum number of spikelets of 22.8 and 23.0 per spike, respectively. Grom achieved a maximum of 22.5 spikelets per spike, while Yr10/6*Avocet S and Yr15/6*Avocet S recorded lower maximums of 19 and 18 spikelets per spike, respectively.

Number of Grains per Spike: The highest average number of grains per spike among the parental samples was observed in the Grom variety with a mean of 53.04 ± 1.22 grains, which once again confirms the high yield potential of this variety. Among the BC_2_F_2_ generations crossed with Grom, the Grom-Yr10 sample showed an even higher result than Grom with an average grain number of 55.36 ± 0.94. Grom-Yr15 had a result close to Grom with 52.78 ± 0.81 grains. The donor varieties—Yr10/6*Avocet S and Yr15/6*Avocet S—had average indicators of 45.81 ± 1.28 and 46.16 ± 0.98 grains, respectively, which were significantly lower than the hybrid samples and the Grom variety. In terms of the maximum number of grains, the BC_2_F_2_ (Grom-Yr10) sample had the highest indicator with 67 grains. The maximum value in Grom was 66 grains, in BC_2_F_2_ (Grom-Yr15) 62.8 grains, and in donor varieties 62 grains (Grom-Yr10) and 64 grains (Grom-Yr15), respectively.

## 3. Discussion

This research successfully integrated the *Yr10* and *Yr15* genes into the high-yielding wheat cultivar Grom using marker-assisted backcrossing to improve resistance to the yellow rust pathogen. The cultivar Grom served as both the recipient and recurrent parent, while Yr10/6*Avocet S and Yr15/6*Avocet S lines functioned as donor lines. The strategy led to the successful development of F_1_ and BC_1_F_1_ hybrids, followed by the production of BC_2_F_3_ ABLs.

The introgression of *Yr10* and *Yr15* was facilitated using tightly linked SSR markers Xpsp3000 (1.2 cM from *Yr10*) and Barc008 (3.9 cM from *Yr15*), both located on chromosome 1BS. Marker-assisted background selection, in conjunction with phenotypic screening, enabled efficient recovery of the Grom genetic background across the BC_1_F_1_, BC_2_F_1_, and BC_2_F_2_ generations.

In the present study, the Barc008 amplified distinct alleles: 245 bp in the resistant parent (Yr15/6*Avocet S) and 210 bp in the susceptible parent (Grom), which aligns with the findings reported by Padhy et al. [32]. The literature reveals considerable variation in the allele sizes for Barc008 in different resistance/susceptible genotypes across different genotypes: 250/280 bp [33], 260/280 bp [27], 185/230 bp [21], 221/257 bp [34], 221/- bp [35], 245/210 bp [32], and 190/230 bp [36]. These differences highlight the genetic diversity and potential environmental or epistatic effects of that marker’s expression. Such variation, while presenting challenges in standardization, also represents an opportunity for plant breeders and geneticists. On one hand, it requires careful consideration; on the other hand, it also expands opportunities for fine-mapping resistance loci and enhancing the precision of molecular breeding.

Phenotyping screening confirmed the effectiveness of the ABLs: while the recurrent parent (Grom) showed susceptibility to yellow rust, the donor parents (Yr10/6*Avocet S and Yr15/6*Avocet S) and all selected BC_2_F_2_ ABLs demonstrated complete resistance, confirming the successful introgression of *Yr10* and *Yr15*.

The Xpsp3000 marker, associated with *Yr10*, produced a 260 bp band in Avocet S6/Yr10 (resistant parent, donor) and a 240 bp band in Grom (susceptible parent, recipient), which is consistent with the findings reported by Mukhtar et al. [21], and Islam et al. [36].

Agronomic evaluations revealed that Grom maintained superior yield traits, such as spike length and grain weight per spike. Notably, BC_2_F_2_ lines, particularly those derived from Grom-Yr10, closely resembled Grom in these traits, indicating minimal yield penalty from gene introgression. By contrast, both donor parents (Yr10/6*Avocet S and Yr15/6*Avocet S) exhibited inferior performance, highlighting a typical trade-off between resistance and productivity. These findings validate the efficacy of MABC in pyramiding disease resistance genes while preserving elite agronomic traits, reinforcing the potential of developed ABLs for future cultivar development in yellow rust-prone environments.

## 4. Materials and Methods

The experiments were conducted between 2018 and 2022 at the experimental field of the Institute of Genetics and Plant Experimental Biology (IGPEB) of the Academy of Sciences of the Republic of Uzbekistan (Kibray, Latitude 41°22′05.3″ N and Longitude 69°24′17.9″ E, Tashkent region).

### 4.1. Plant Materials and Breeding Scheme

**Figure 4:**
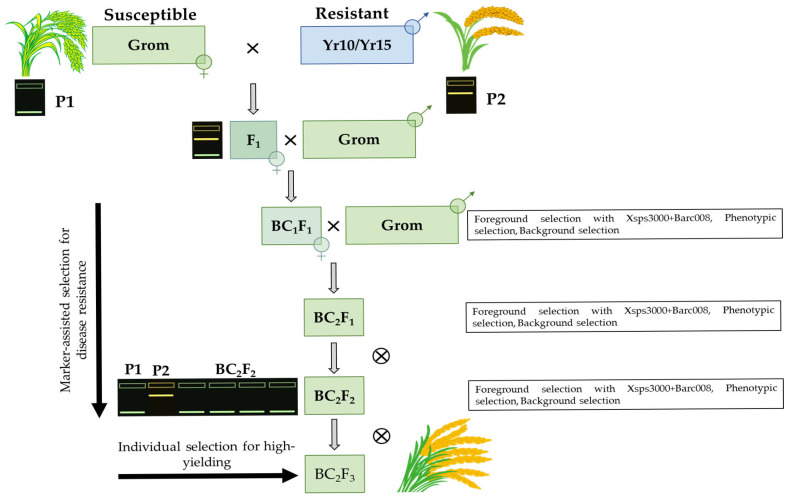
Schematic representation of marker-assisted backcrossing for introgression of yellow rust resistance genes into Grom cultivar.

In this study, we used bread wheat accessions with varying levels of yellow rust resistance in a Marker-Assisted Selection (MAS) program. We used the yellow rust-resistant Yr10/6*Avocet S and Yr15/6*Avocet Sisogenic lines, carrying the *Yr10* and *Yr15* resistance genes respectively, as donor genotypes. A high-yielding bread wheat cultivar, Grom, widely cultivated in Uzbekistan, served as a recipient parent (Figure 4). The Grom cultivar is susceptible to yellow rust compared to the donor parents. The wheat cultivar Morocco, lacking the desired marker alleles associated with *Yr10* and *Yr15* genes, was used as a negative control during the MAS breeding process.

We carried out crosses between the Grom × Yr10/6*Avocet S and Grom × Yr15/6*Avocet S isogenic lines. Following this, F_1_ hybrids were subjected to successive backcrossing up to the BC_2_F_3_ generations, resulting in segregating populations focused on the specific targeted QTL regions. Only heterozygotes were selected with the desired alleles for the subsequent breeding process. The backcross hybrids were developed up to the BC_2_F_3_ generations. Hybrid genotypes with resistance genes and favorable morphological and agronomic traits were individually selected from the BC_2_F_3_ population plants based on their homozygous marker allele and trait improvement, as described above.

### 4.2. Assessment of Yellow Rust Resistance

To evaluate the resistance, plant samples were inoculated artificially with *P. striiformis* urediniospores. The susceptible Morocco cultivar was planted as a border around the experimental field to ensure uniform infection. The inoculum containing the urediniospores of *P. striiformis* was prepared immediately before use in a solution of distilled water and Tween-20. The spore suspension was prepared at a concentration of 6 × 10^5^ spores/mL, with approximately 0.6 mL applied per plant.

Disease assessment data, including infected leaf surface (severity) and infection type (IT), were recorded 14–15 days after inoculation with *P. striiformis*, when the control plants reached maximum sporulation. The severity of *P. striiformis* infection was visually scored using the modified Cobb’s scale [31], which quantifies the percentage of leaf area affected by rust on a scale from 1% to 100%. Infection type (IT) was also categorized into five classes based on host response: 0 (immune), R (resistant), MR (moderately resistant), MS (moderately susceptible), and S (susceptible), following the classification by Peterson et al. [31].

### 4.3. Genomic DNA Isolation and PCR Assay

The genomic DNA was extracted from the fresh leaf tissues of wheat genotypes using the modified CTAB method, as previously described in earlier studies [9]. The DNA was diluted to a working concentration of 25 ng/μL and stored at −20 °C.

The polymerase chain reaction (PCR) was conducted in 10 μL volumes, containing 1.0 μL of 10× PCR buffer with 1.5 mM MgCl_2_, 0.5 μL of a 25 mM dNTPs mixture, 0.5 μL (50 ng/μL) of each primer, 0.5 μL (25 ng/μL) of template DNA, 0.5 U of Taq DNA polymerase (Syntol, Moscow, Russia), and 7.0 μL DNase/RNase-Free Distilled Water.

In this study, 86 SSR markers associated with yellow rust (YR) resistance were ini-tially screened to detect DNA polymorphisms between the parent cultivars and isogenic lines, of which 35 markers showed polymorphism, which were used for background selection (Supplementary Table S1). Phenotypic and genotypic evaluations of wheat samples for yellow rust resistance were compared in the field. Among the markers, Xpsp3000 (linked to the *Yr10* gene) and Barc008 (linked to the *Yr15* gene) demonstrated the closest association with phenotypic resistance. Therefore, these two markers were used for selecting the most promising resistant samples.

### 4.4. Statistical Analysis

Using markers, we selected samples resistant to yellow rust, measured their morpho-economic traits and performed statistical analysis. Statistical analyses were performed using the OriginPro 8.5 2022 software package [12].

The statistical analysis of traits contributing to yield was determined using the Ken Sayre method [37]. Productivity-related traits such as spike length (cm), spike weight (g), grain weight per spike (g), number of spikelets per spike, and number of grains per spike were evaluated at the physiological maturity stage. For each genotype (BC_2_F_2_, Yr10/6*Avocet S, Yr15/6*Avocet S, and Grom), measurements were taken from ten randomly selected plants. Spike length was measured using a ruler from the base to the tip of the spike (excluding awns). The collected data were subjected to statistical analysis to calculate mean values and standard deviations.

The standard deviation (SD), standard error (SE), and coefficient of variation (CV%) were calculated using standard statistical methods based on 10 measurements.

## 5. Conclusions

This study successfully demonstrates the introgression of the *Yr10* and *Yr15* yellow rust resistance genes into the high-yielding wheat cultivar Grom through a marker-assisted backcrossing approach. The use of tightly linked SSR markers Xpsp3000 (*Yr10*) and Barc008 (*Yr15*) enabled precise selection during foreground and background screening. Initial hybridizations produced heterozygous F_1_ plants, followed by backcrossing and selfing to generate BC_2_F_2_ progenies exhibiting homozygosity for the target resistance loci.

Phenotypic evaluations confirmed that the developed ABLs were resistant to yellow rust—*Puccinia striiformis* f. sp. *tritici*, with infection types consistent with the donor parents, while maintaining the superior yield-related traits of Grom. Agronomic analyses revealed that key traits, such as spike length, spike weight, and grain number per spike remained comparable to Grom, particularly in BC_2_F_2_ lines derived from Grom-Yr15, indicating minimal yield penalty associated with gene introgression.

The selection of elite BC_2_F_2_ individuals led to the generation of homozygous BC_2_F_3_ ABLs combining robust resistance and high productivity. These lines represent valuable germplasm for wheat improvement in yellow rust-prone environments and exemplify the power of integrating molecular markers with conventional breeding for durable disease resistance.

## Figures and Tables

**Table 1:** Number of gene-positive plants identified in each backcross generation.

Donor Parent	Target Gene	Specific Marker	Recipient/Recurrent Parent	Generation	No. of Plants Screened with DNA Marker	No. of Plants Carrying *Yr* Genes	
						Heterozygous	Homozygous
Yr10/6*Avocet S	*Yr10*	Xpsp3000	Grom	BC_1_F_1_	17	8	-
				BC_2_F_1_	124	57	-
				BC_2_F_2_	99	49	23
Yr15/6*Avocet S	*Yr15*	Barc008		BC_1_F_1_	32	15	-
				BC_2_F_1_	110	51	-
				BC_2_F_2_	127	67	29

**Table 2:** Yield-related traits of backcross lines with parental samples.

Plant Samples	Limit (Min–Max)	Mean	SD	SE	CV%
Spike length, cm					
Yr10/6*Avocet S	7.5–11.0	9.08	0.87	0.17	9.62
Yr15/6*Avocet S	8.0–11.0	9.31	0.65	0.10	7.0
Grom	9.0–13.0	10.97	1.02	0.21	9.36
BC_2_F_2_ (Grom-Yr10)	7.0–13.0	10.58	1.13	0.23	10.75
BC_2_F_2_ (Grom-Yr15)	9.2–11.9	10.16	0.69	0.12	6.84
Spike weight, g					
Yr10/6*Avocet S	1.59–2.62	2.06	0.21	0.04	10.56
Yr15/6*Avocet S	1.56–2.89	2.21	0.31	0.04	14.17
Grom	2.0–3.18	2.57	0.35	0.07	13.17
BC_2_F_2_ (Grom-Yr10)	2.01–3.23	2.52	0.33	0.07	13.34
BC_2_F_2_ (Grom-Yr15)	1.94–2.92	2.50	0.31	0.05	12.65
Grain weight per spike, g					
Yr10/6*Avocet S	1.38–2.30	1.68	0.24	0.04	14.28
Yr15/6*Avocet S	1.16–2.36	1.70	0.29	0.04	17.08
Grom	1.66–2.97	2.06	0.38	0.08	18.87
BC_2_F_2_ (Grom-Yr10)	1.65–2.82	2.05	0.32	0.06	15.88
BC_2_F_2_ (Grom-Yr15)	1.59–2.41	2.04	0.28	0.05	13.93
No. of spikelets per spike					
Yr10/6*Avocet S	14.0–19.0	16.14	1.32	0.25	8.18
Yr15/6*Avocet S	14.0–18.0	16.28	1.17	0.18	7.21
Grom	19.0–22.5	20.17	0.99	0.20	4.93
BC_2_F_2_ (Grom-Yr10)	17.0–23.0	19.88	1.51	0.31	7.59
BC_2_F_2_ (Grom-Yr15)	16.0–22.8	19.30	1.56	0.29	8.12
No. of grains per spike					
Yr10/6*Avocet S	37.0–62.0	45.81	6.69	1.28	14.61
Yr15/6*Avocet S	37.0–64.0	46.16	6.40	0.98	13.86
Grom	47.0–66.0	53.04	5.88	1.22	11.08
BC_2_F_2_ (Grom-Yr10)	48.0–67.0	55.36	5.33	0.94	9.64
BC_2_F_2_ (Grom-Yr15)	46.6–62.8	52.78	4.38	0.81	8.29

## Data Availability

The original contributions presented in this study are included in the article/Supplementary Materials. Further inquiries can be directed to the corresponding authors.

## Supplementary Materials

The supporting information can be downloaded at https://www.mdpi.com/article/10.3390/ijms26157603/s1.
