# Supplementary material for: Development of Yellow Rust-Resistant and High-Yielding Bread Wheat (Triticum aestivum L.) Lines Using Marker-Assisted Backcrossing Strategies
Source: Int J Mol Sci. 2025 Aug 6;26(15):7603. doi: 10.3390/ijms26157603 (PMC12347614; doi:10.3390/ijms26157603)
Supplement: Supplementary file 1 [file ijms-26-07603-s001.zip › ijms-3684931-supplementary.pdf]

Supplementary Table S1: Polymorphic SSR markers, and their polymorphism information content (PIC) and heterozygosity (He) values

Marker	Forward (5'-3')	Reverse (3'-5')	PIC	HE	Chr. No.	References
Barc172	GCGAAATGTGATGGGGTTTATCTA	GCGATTTGATTTAACTTTAGCAGTGAG	0,666	0,592	7D	Somers et al., 2004
Xgwm111	TCTGTAGGCTCTCTCCGACTG	ACCTGATCAGATCCCACTCG	0,666	0,592	7D	Zahravi et al., 2003
Xgwm106	CTGTTCTTGCGTGGCATTAA	AATAAGGACACAATTGGGATGG	0,666	0,592	1D	Roder et al., 1998
Xgwm635	TTCCTCACTGTAAGGGCGTT	CAGCCTTAGCCTTGGCG	0,666	0,592	7D	Roder et al., 1998
Gwm332c	AGCCAGCAAGTCACCAAAAC	AGTGCTGGAAAGAGTAGTGAAGC	0,44	0,345	7A	Roder et al., 1998
Gwm340	GCAATCTTTTTTCTGACCACG	ACGAGGCAAGAACACACATG	0,375	0,304	3B	Imtiaz et al., 2001
Xgwm77	ACAAAGGTAAGCAGCACCTG	ACCCTCTTGCCCGTGTTG	0,625	0,554	3B	Roder et al., 1998
Xgwm18	TGGCGCCATGATTGCATT ATCTTC	GGT TGC TGA AGA ACCTTA TTT AGG	0,625	0,554	1B	Roder et al., 1998
Xgwm146	CCAAAAAAACTGCCTGCATG	CTCTGGCATTGCTCCTTGG	0,625	0,554	7B	Roder et al., 1998
Xgwm294	GGATTGGAGTTAAGAGAGAACCG	GCAGAGTGATCAATGCCAGA	0,625	0,554	2A	Roder et al., 1998
Xgwm344	CAAGGAAATAGGCGGTAACT	ATTTGAGTCTGAAGTTTGCA	0,642	0,569	2B	Somers et al., 2004
Wmc797	CGAAACCCTAGATGAAGC	ACACAACCACAGGTGAGTTGTTCT	0,625	0,554	2D	Somers et al., 2004
Wmc824	CCGATGAACTTAAAAGTACCACCTG	CATGGATTGACACGATTGGC	0,625	0,554	7D	Somers et al., 2004
Wmc382	CATGAATGGAGGCACTGAAACA	CCTTCCGGTCGACGCAAC	0,625	0,554	2A	Somers et al., 2004
Gwm140	ATGGAGATATTTGGCCTACAAC	CTTGACTTCAAGGCGTGACA	0,375	0,304	1B	Roder et al., 1998
Gwm437	GATCAAGACTTTTGTATCTCTC	GATGTCCAACAGTTAGCTTA	0,625	0,554	7D	Zahravi et al., 2003
Barc0187	GTGGTATTTCAGGTGGAGTTGTTTTA	CGGAGGAGCAGTAAGGAAGG	0,625	0,554	1B	Wen et al., 2008
Gwm295	GTGAAGCAGACCCACAACAC	GACGGCTGCGACGTAGAG	0,375	0,304	7D	Roder et al., 1998
Wmc149	ACAGACTTGGTTGGTGCCGAGC	ATGGGCGGGGGTGTAGAGTTTG	0,625	0,554	2B,2A	Somers et al., 2004
Barc0101	GCTCCTCTCACGATCACGCAAAG	GCGAGTCGATCACACTATGAGCCAATG	0,625	0,554	2B	Somers et al., 2004
Wmc656	AAGTAGGCGAGCGTTGT	TTTCCCTGGCGAGATG	0,625	0,554	3D	Somers et al., 2004
Gwm011	GGATAGTCAGACAATTCTTGTG	GTGAATTGTGTCTTGTATGCTTCC	0,625	0,554	1B	Roder et al., 1998
Barc0008	GCGGGAATCATGCATAGGAAAACAGAA	GCGGGGCGAAACATACACATAAAAACA	0,625	0,554	1BS	ElBasyoni et al., 2021
Wmc473	TCTGTTGCGCGAAACAGAATAG	CCCATTGGACAACACTTTCACC	0,6	0,533	6B	Somers et al., 2004
Xgwm501	GGCTATCTCTGGCGCTAAAA	TCCACAAACAAGTAGCGCC	0,625	0,554	2BL	Rani et al., 2019
Xgwm311	TCACGTGGAAGACGCTCC	CTACGTGCACCACCATTTTG	0,625	0,554	2A	Roder et al., 1998
Gwm413	TGCTTGTCTAGATTGCTTGGG	GATCGTCTCGTCCTTGGCA	0,625	0,554	1B	Roder et al., 1998
Xgwm642	ACGGCGAGAAGGTGCTC	CATGAAAGGCAAGTTCGTCA	0,625	0,554	1D	Somers et al., 2004
Barc76	ATTCGTTGCTGCCACTTGCTG	GCGCGACACGGAGTAAGGACACC	0,625	0,554	6B	Somers et al., 2004
Gwm161	GATCGAGTGATGGCAGATGG	TGTGAATTACTTGGACGTGG	0,625	0,554	4A	Lowe et al., 2011
Xgwm273	ATTGGACGGACAGATGCTTT	AGCAGTGAGGAAGGGGATC	0,625	0,554	1BS	Waris et al., 2023
Wmc407	GGTAATTCTAGGCTGACATATGCTC	CATATTTCCAAATCCCCAACTC	0,625	0,554	2A	Somers et al., 2004

Wmc477	CGTCGAAAACCGTACACTCTCC	GCGAAACAGAATAGCCCTGATG	0,625	0,554	2B	Somers et al., 2004
Xpsp3000	GCAGACCTGTGTCATTGGTC	GATATAGTGGCAGCAGGATACG	0,625	0,554	1BS	ElBasyoni et al., 2021
Barc349	CGAATAGCCGCTGCACAAG	TATGCATGCCTTTCTTTACAAT	0,625	0,554	2B	Lowe et al., 2011
